# Sequencing and phylogenetic analysis of the gp51 gene from Korean bovine leukemia virus isolates

**DOI:** 10.1186/s12985-015-0286-4

**Published:** 2015-04-15

**Authors:** EunJung Lee, Eun-Ju Kim, Ha-Kyung Joung, Bo-Hye Kim, Jae-Young Song, In-Soo Cho, Kyoung-Ki Lee, Yeun-Kyung Shin

**Affiliations:** Viral Disease Division, Animal and Plant Quarantine Agency, 175 Anyangro, Anyang, Gyeonggido 430-757 Republic of Korea; Veterinary Drugs and Biologics Division, Anyang, 430-757 Gyeonggido Republic of Korea; Animal Disease Diagnostic Division, Animal and Plant Quarantine Agency, Anyang, 430-757 Gyeonggido Republic of Korea

**Keywords:** Bovine leukemia virus (BLV), *env* gene, gp51, Phylogenetic analysis, Korea

## Abstract

**Background:**

Bovine Leukemia virus (BLV) infection of cattle has been reported in Korea for more than three decades. However, to date, there have been few studies regarding Korean BLV since 1980s. Thus, the purpose of this study is to perform a diagnosis and molecular characterization of BLV strains circulating in Korea and to estimate genetic diversity of different genotypes of BLV.

**Method:**

To investigate the distribution of BLV variants in the world and assess the evolutionary history of Korean BLV isolates, a comprehensive molecular analysis of the BLV *env* gp51 gene was conducted using recent worldwide BLV isolates. The isolates included 50 samples obtained from two cattle farms in southeastern Korea in 2014.

**Results:**

Sequence and phylogenetic analyses of partial 444-nt fragment sequences and complete gp51 sequences of BLV revealed eight distinct genotypes of BLV showing geographic distribution of the world. Most Korean BLV isolates were found to belong to genotype 1 which is a major genotype prevailed throughout the world, and only four isolates from one farm were classified as genotype 3 related to the US and Japan isolates. Analysis of amino acids of Korean BLV isolates showed several sequence substitutions in the leader peptide, conformational epitope, and neutralizing domain regions. The observations suggest the possibility of affecting on viral infectivity and formation.

**Conclusion:**

Korean BLV isolates showed the close relationship to genotype 1 and 3. Further study to identify the diversity of BLV circulating in Korea is necessary with samples collected nationwide because this study is the first report of BLV genotype 3 being in circulation in Korea.

**Electronic supplementary material:**

The online version of this article (doi:10.1186/s12985-015-0286-4) contains supplementary material, which is available to authorized users.

## Background

Bovine Leukemia virus (BLV) belongs to the genus *Deltaretrovirus* of the *Retroviridae* family and is the causative agent of enzootic bovine leukosis (EBL), a disease that results in economic loss for the cattle industry [1]. BLV infected cattle are asymptomatic; 30 ~ 70% of infected animals develop persistent lymphocytosis (PL) and 0.1 ~ 10% develop lymphoid tumors [2-4]. BLV infection can be found worldwide, but many European countries have successfully eradicated EBL in recent years [1,4-14].

BLV has an envelope (Env) protein complex that is composed of surface glycoprotein (SU) subunits, which are anchored to virions by their association with transmembrane (TM) protein subunits [15]. The envelope glycoproteins of BVL play a crucial role in the virus life cycle; envelope proteins are responsible for cellular tropism because they contain the recognition site for the cell surface receptor required for virus entry and are the natural target for neutralizing antibodies [16].

Recent phylogenetic analysis of BLV *env* genes showed that this virus could be classified into 8 or more genotypes [17,18]. EBL was first reported by the serological method in Korea in the 1980s [19], and recently, its prevalence in dairy cattle was found to be over 50% [20-23]. To date, there has been no reports of the BLV genotype circulating in Korea since partial *env* gene sequences that belonged to genotype 1 were deposited in 2009 [24]. It is known that some of the genotype groupings correlate with the geographical origin of the strain [16,25-34], but with the increased movement of cattle and people around the world, a variety of genotypes have been reported in places where only a specific genotype was dominant in the past [17,33]. In Japanese studies, it was reported that many genotypes of BLV (genotypes 1 ~ 5) co-circulated nationwide [35] with genotype 1 being predominant and genotypes 3 and 5 being widely distributed [36].

This study was performed to identify the genetic diversity of BLV in Korean cattle. The gp51 sequence in 50 BLV isolates was analyzed in our study.

## Methods

### Samples information

A total of 185 whole blood samples were collected from dairy cattle on two farms (119 from farm A and 66 from farm B) in southeast Korea. Farm A is located in Gyeongsangbuk-do and farm B in Gyeongsangnam-do. The samples were collected between April and July 2014.

### Viral DNA extraction

Peripheral blood lymphocytes (PBLs) were isolated from whole blood samples and stored at −70°C until further use. PBLs isolation was performed using Luecosep tube (Greiner Bio-One Gmbh, Frickenhausen, Germany) according to the manufacturer’s protocol. Isolated PBLs were resuspended in 1 ml of PBS. Viral DNA was extracted from 200 μL of PBL solution with QIAamp DNA Mini Kit (QIAGEN, Hilden, Germany) following the manufacturer’s recommendations. DNA samples were stored at −20°C until further use.

### PCR amplification of the *env* gene fragment

BLV gp51 gene amplification for diagnosis was carried out using primers sets previously described by Fechner et al. [37]. The PCR reaction was performed using a Maxime PCR PreMix Kit (iNtRON Biotechnongy, Gyeonggi-do, Korea). The first PCR was performed using a 20-μL reaction volume. For one reaction, the assay was optimized to contain 1× PCR buffer, 1 μL of DNA (~1 μg), 1 μL of each of the env-specific primers BLV-env-1 (5′-TCTGTGCCAAGTCTCCCAGATA-3′) and BLV-env-2 (5′-AACAACAACCTCTGGGAAGGG-3′) (20 pmol/μL), 2.5 mM of each dNTP, 2.5 units of *i*-Taq polymerase. The reaction parameters were 2 minutes at 94°C, followed by 30 cycles of 30 seconds at 95°C, 30 seconds at 58°C and 60 seconds at 72°C and 4 minutes at 72°C. The nested PCR was performed using a 20-μL reaction volume. For one reaction, the assay was optimized to contain 0.5 μL of PCR product of the first PCR, 1× PCR buffer, 0.5 μL of each of the env-specific primers BLV-env-3 (5′-CCCACAAGGGCGGCGCCGGTTT-3′) and BLV-env-4 (5′-GCGAGGCCGGGTCCAGAGCTGG-3′) (20 pmol/μL), 2.5 mM of each dNTP, 2.5 units of *i*-Taq polymerase. The PCR reactions were performed using a T3000 thermocycler 48 (Biometra, Rundolf wissell-str, Germany) with parameters of 2 minutes at 94°C, followed by 30 cycles of 30 seconds at 95°C, 30 seconds at 58°C and 60 seconds at 72°C and 4 minutes at 72°C. The PCR products were analyzed by electrophoresis on a 1.5% agarose gel containing ethidium bromide (1 μg/mL).

The Gp51 full gene (903 bp) for sequencing was amplified using HotStar Hifidelity DNA Polymerase (QIAGEN, Hilgen, Germany) and a primer set (BLV-F-4807; CTGGCGTTTGYTGAAGGCCTT, BLV-gp51-R; ACTACGTCTGACCCGGGTA) following the manufacturer’s recommendation. The reaction mixture contained 3 μL of template, 1 μL of HotStar Hifidelity DNA Polymerase, 10 μL of 5X PCR buffer, 1 μL of each primer (10 pmol/μL) and 34 μL of distilled H2O. The PCR reaction was performed on a T3000 thermocycler 48 (Biometra, Rundolf wissell-str, Germany) with parameters of 5 min at 95°C, followed by 40 cycles of 15 s at 94°C, 60 s at 58°C, and 60 s at 72°C and 10 min at 72°C. The PCR products were analyzed by electrophoresis on a 1.5% agarose gel containing ethidium bromide (1 μg/mL).

### Sequencing of the gp51

The gp51 PCR products were purified using a PCR purification kit (QIAGEN, Hilgen, Germany). The purified amplicons were cloned into the pDrive vector (QIAGEN, Hilgen, Germany) and sequenced by a local company (Macrogen Inc. Seoul, Korea). The sequences were analyzed using the DNAstar program (DNASTAR Inc., Ver. 5.0).

### Phylogenetic analysis

To elucidate the genetic relationships between Korean and worldwide BLV isolates, currently available BLV *env* sequences were downloaded from the GenBank database and combined with 17 unique Korean BLV *env* fragment sequences. After online alignment using MAFFT v. 7 (http://mafft.cbrc.jp/alignment/server/) [38], sequences with too many missing characters were manually excluded. To consider all of the possible genotypes of the worldwide BLV isolates, two different datasets were generated for phylogenetic analyses: partial gp51 (444 bp), ranging from 93 to 240 amino acids, and complete gp51 (903 bp), ranging from 1 to 301 amino acids [39]. To estimate the genetic divergence of the gp51 gene, the Kimura-2-parameter (K2P) [40] of the genetic distance was calculated using MEGA v.5 [41] for both intra- and inter-genotype distances. To compare the gp51 amino acid sequences from Korean to other isolates from other countries, protein sequences based on a BLV reference sequence (i.e., K02120 and AY995174) were obtained by MEGA.

For accurate and robust phylogenetic analyses of the BLV gp51 sequence, the Maximum likelihood (ML) and Bayesian Inference (BI) approaches were performed. To determine the most appropriate nucleotide substitution models, jModelTest v.2.1.4 [42] was used among 88 candidates on a BioNJ tree, under the Akaike Information Criterion (AIC), corrected AIC(c), and Bayesian Information Criterion (BIC). The closest model available in the corresponding ML and Bayesian analyses were chosen for each dataset.

For the ML analyses, GARLI 2.01 [43], based on genetic algorithms for the ML search, and RAxML 7.4.2 [44], implemented in raxmlGUI v.1.3 [45], were used to compare the phylogenetic results. Because RAxML only allows the most complex GTR (General Time Reversible) substitution model but GARLI is available for the standard nested submodels of the GTR, the following models were selected after model estimation for each dataset: GTR + I (proportion of the invariable sites) (903 bp) and GTR + G (Gamma distribution of the rates among sites) (444 bp) for RAxML and TPM3uf + I (903 bp) and TPM2 + G (444 bp) for GARLI. Bootstrap supports were calculated using 1,000 bootstrap replicates for the four datasets, and the bootstrap trees were summarized with a 50% majority rule consensus tree by the SumTrees script (v.3.3.1) implemented in DendroPy v.3.10.1 [46].

For the Bayesian analyses, two programs were used. The first was MrBayes v.3.2.2 [47], but such analyses have been reported to produce biased posterior probabilities in certain cases (e.g., the “star-tree paradox” [48]). To address this issue, Phycas v.1.2.0 [49] was used to as a second approach for the polytomy prior [50]. As the best-fit models, HKY (Hasegawa, Kishino and Yano) + I (903 bp) and HKY + G (444 bp) were specified for each dataset in MrBayes. Two runs with four chains were carried out simultaneously for 10 million generations, and the trees were sampled every 100 generations. The convergence diagnostics were evaluated when the average standard deviation of the split frequencies of the two runs was less than 0.01. In Phycas, the same substitution models of MrBayes were used for each dataset, but with a 1 million generation. Tree samples prior to reaching a stationary posterior distribution were discarded, and the remaining samples were summarized with SumTrees.

The phylogenetic trees are rooted on genotype 5, which includes isolates from Central and South America for a comparison to previous studies [17,18,33,51].

## Results

### Diagnosis and identification of Korean BLV isolates

**Figure 1:**
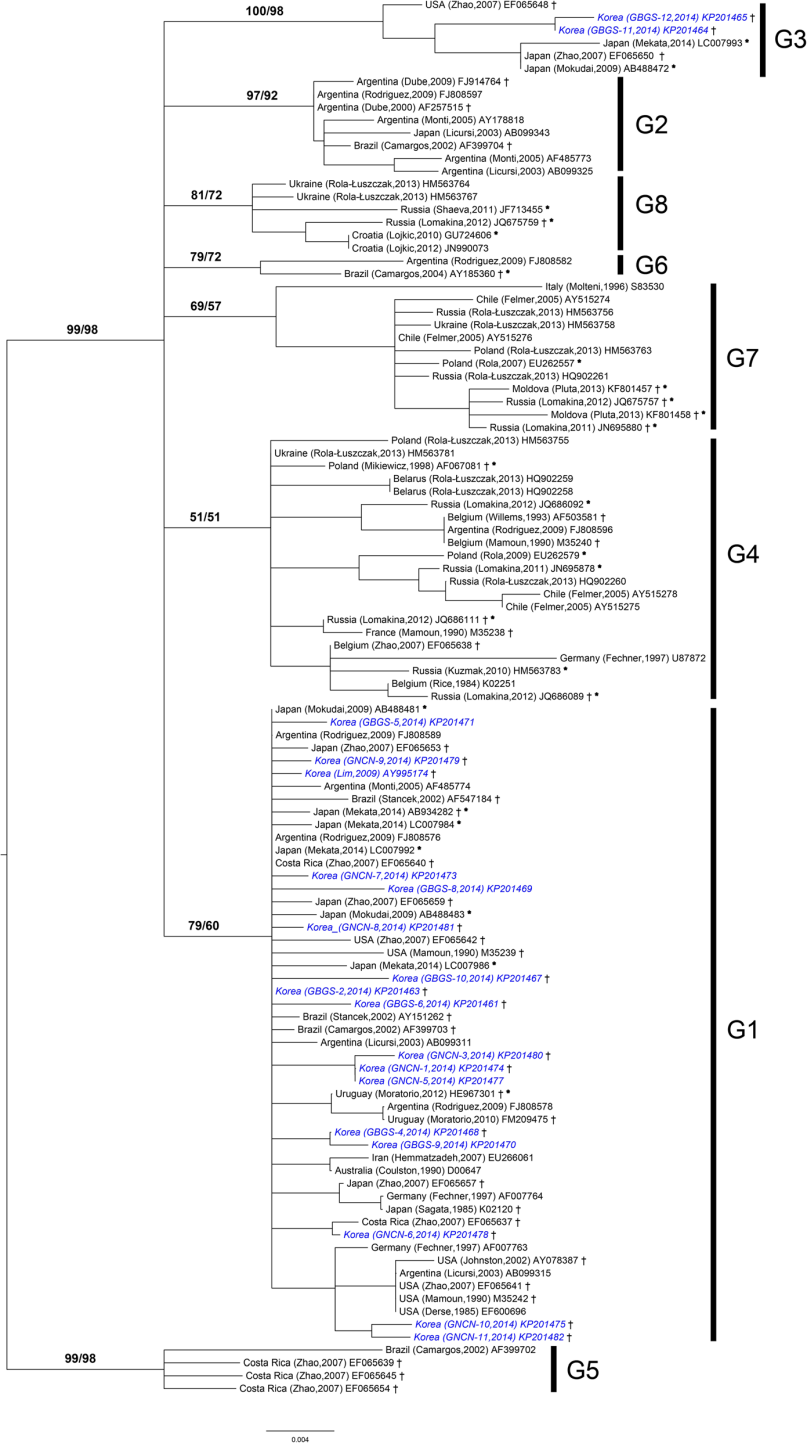
Maximum-likelihood phylogenetic tree of the partial gp51 gene (444 bp) sequences from different geographic regions. Korean isolates are shown in blue bold-italic names. The remaining isolates in the tree are denoted by country of origin, author with published date (or directly submitted date), and accession number. Genotypes 1 through 8 are indicated by vertical lines with the symbol ‘G’. Two numbers at the branches indicate ML bootstrap support values (RaxML/Garli). An asterisk indicates unpublished or direct submission to GenBank sequences. + denotes that the selected sequences were used for the complete gp51 analyses. *Means that the sequences have not yet been investigated by a published phylogenetic analysis. The tree is rooted on genotype 5.

**Figure 2:**
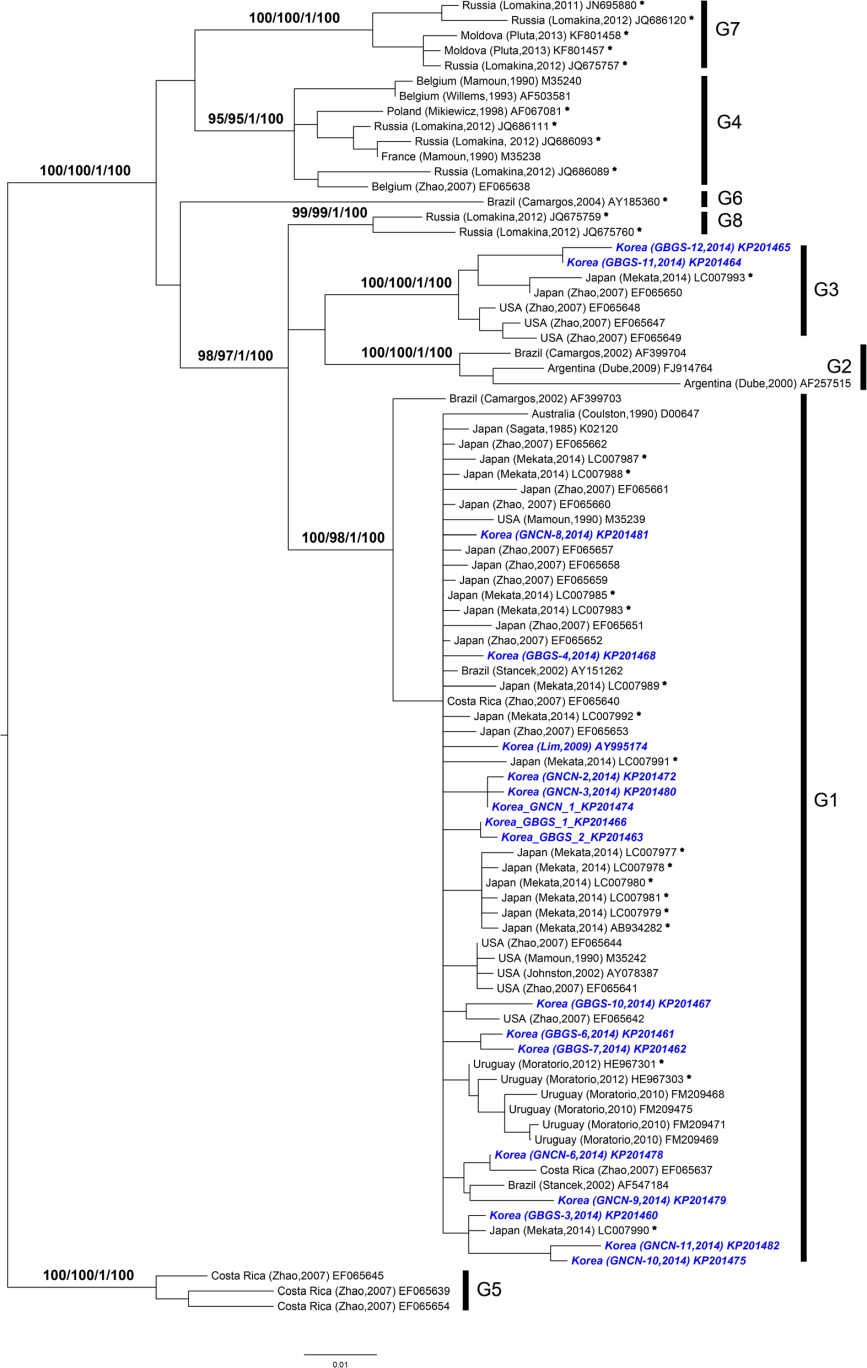
A phylogenetic tree based on the full-length gp51 gene sequences of BLV isolates. Korean isolates are shown in blue bold-italic names. The remaining isolates in the tree are denoted by country of origin, author with published date (or directly submitted date), and accession number. Numbers at nodes indicate bootstrap support values for Maximum likelihood (RaxM:1, Garli:2) and posterior probabilities for Bayesian inference (MrBayes:3 (0–1), Phycas:4 (0–100)). *Means that the sequences have not yet been investigated by a published phylogenetic analysis. The tree is rooted on genotype 5.

A total of 185 samples (119 samples from farm A and 66 samples from farm B) were analyzed, and 78 samples (42.2%) were positive for BLV *env* DNA, as detected by nested PCR for a 444-bp fragment (37 samples out of 119 in farm A and 41 out of 66 in farm B) (Table 1). Another PCR reaction to amplify the full gp51 gene was also performed. Nineteen out of 37 positive samples from farm A and 31 out of 41 positive samples from farm B were successfully amplified. As a result of the phylogenic analysis based on the Korean isolates, the genotypes from farm A was divided into two groups, genotype 1 and genotype 3 (Figures 1 and 2). Out of the 19 samples analyzed from farm A, 15 were genotype 1 (78.9%) and 4 were genotype 3 (21.1%). We found that all 31 isolates from farm B belong to genotype 1. Of all of the cloned gp51 sequences, 46 samples were genotype 1 (92%) and 4 samples were genotype 3 (8%). All of the sequences of BLV gp51 obtained in this study can be found in Additional file 1: Table S1 and are deposited in the GenBank databases with accession numbers KP20146-KP201482.

**Table 1:** **Identification of Korean BLV isolates**

	**No. cattle tested**	**No. positive in nested PCR**	**No. gp51 gene amplified**	**Genotype**	
				**G1**	**G3**
Farm A	119	37	19	15	4
Farm B	66	41	31	31	-
Total (%)	185	78 (42.2)	50	46 (92)	4 (8)

### Genotyping of the BLV gp51 gene based on global phylogenetic analyses

ML and BI trees based on the complete gp51 gene showed congruent topologies. The existence of eight genotypes of BLV was highly supported by high bootstrap and posterior probability values of more than 95 and 0.99, respectively (Figure 2). Phylogenetic analyses based on the partial 444 bp of gp51 also supported the distinct eight genotypes of BLV, which was previously described [17,18]. However, there were some discrepancies in the genotypic phylogenetic relationships between the ML and Bayesian analyses (Figure 1, Additional file 1: Figure S1). The use of different substitution models had little effect on the overall analyses of the genotype separation, regardless of the datasets. Supporting values for each node were relatively lower in ML than BI (Figures 1 and 2, Additional file 1: Figure S1).

Evolutionary relationships among the genotypes showed some correlation between the genotypes and geographic affinities. For instance, genotypes 7 and 4, mostly from European countries, have a close relationship with a node receiving less than 80% support, except for MrBayes (74/67/0.9/68: data not shown on the tree). Compared to the G4-G7 relationship, genotypes 2 and 3 are more closely related to each other, as has been previously described [17,18,33,36,51,52], with 88/85/0.92/67. However, the phylogenetic relationships between these groups were not fully resolved in the partial gp51 analyses.

Worldwide distribution of BLV genotypes based on partial gp51 sequences is summarized in Table 2. Genotype 1 is found in 10 countries (i.e., Korea, Japan, USA, Costa Rica, Argentina, Uruguay, Brazil, Iran, Australia, and Germany) and is the most prevalent genotype worldwide, whereas genotypes 4, 7, and 8 are mainly distributed in Europe and Russia. However, the second most widely distributed genotype, G4, is found more frequently in Europe and America. The prevalent BLV genotypes in Central and South America appear to have more variability, including G1, G2, G4, G5, G6, and G7. Among these, genotypes 5 and 6 were exclusively found in Argentinean, Brazilian, and Costa Rican isolates. Interestingly, genotype 3 isolates from the USA are closely related to those from Korea and Japan.

**Table 2:** **Worldwide geographic distribution of eight BLV genotypes based on 109 sequences of the partial 444-bp fragment of gp51**

**Geographic divisions**	**Country**	**Genotype** ^**1**^								**References** ^**2**^
		**1**	**2**	**3**	**4**	**5**	**6**	**7**	**8**	
East Asia	Korea	1		3						This study, Lim *et al.*(2009) [24]
	Japan	1	2	3						Licursi *et al.(*2003) [34]; Matsumura *et al.*(2011) [36]; Sagata *et al.*(1985) [56]; Zhao & Buehring (2007) [30]
North America	USA	1		3	***4***					Derse *et al.* (1985) [59]; Johnston *et al.*(2002) [15]; Mamoun *et al.* (1990) [16]; Zhao & Buehring (2007) [30]
Central America	Costa Rica	1				5				Zhao & Buehring (2007) [30]
South America	Argentina	1	2		4		6			Dube *et al.* (2009) [53]; Dube *et al.* (2000) [54]; Licursi *et al.*(2003) [34]; Monti *et al.*(2005) [29]; Rodriguez *et al.* (2009) [33]
	Brazil	1	2			5	6	***7***		Camargos *et al.* (2007) [31]; Camargos *et al.* (2002) [32]; D’Angelino *et al.* (2013) [52]; Moratorio *et al.* (2010) [51]
	Chile				4			7		Felmer *et al.* (2005) [26]
	Uruguay	1								Moratorio *et al.* (2010) [51]
Middle East	Iran	1								Hemmatzadeh (2007) [27]
Australia	Australia	1								Coulston *et al.* (1990) [25]
Eastern Europe	Belarus				4					Rola-Luszczak *et al.*(2013) [18]
	Moldova							7		Pluta *et al.* (unpublished)
	Russia				4			7	8	Rola-Luszczak *et al.*(2013) [18]
	Ukraine				4			7	8	Rola-Luszczak *et al.*(2013) [18]
Central Europe	Croatia								8	Balic *et al.*(2012) [17]
	Poland				4			7		Rola-Luszczak *et al.*(2013) [18]
Western Europe	Belgium				4					Mamoun *et al.*(1990) [16]; Rice *et al.* (1984) [55]; Willems *et al.* (1993) [57]; Zhao & Buehring (2007) [30]
	France			***3***	4					Mamoun *et al.* (1990) [16]
	Germany	1			4					Fechner *et al.* (1997) [37]
	Italy							7		Molteni *et al.* (1996) [28]

^1^The number in each column corresponds to each country’s prevalent BLV genotype, and the genotype in bold italic numbers denote that their sequences were excluded for accurate phylogenetic analyses because they were too short (France: M35241), very divergent (Brazil: DQ059417), or of unknown origin (USA: AF033818). However, they are closely related to their corresponding genotypes with a high degree of sequence similarity.

^2^A reference list of sequences used for phylogenetic analyses and genotyping in this study. Unpublished and direct submission to GenBank sequences are marked with an asterisk in the phylogenetic trees (Figures 1 and 2, Additional file 1: Figure S1).

### Genetic variation of the complete gp51 gene

The complete gp51 sequences in this study include 87 isolates with 903 nucleotides, of which, 142 nucleotides were parsimoniously informative. The average evolutionary divergence of the sequence pairs is 2.3%; similarly, 2.5% divergence was observed in the partial 444 bp from 109 sequences. Specifically, the minimum to maximum divergence range was 0.11% to 1.91% for genotype 1 and 0.33% to 1.58% for genotype 3. The estimated average divergence over sequence pairs within and between genotypes is described in Table 3.

**Table 3:** **The average percentage of genetic distance among the main genotypes based on the Kimura-2-parameter** ^*^

	**G1**	**G2**	**G3**	**G4**	**G5**	**G6**	**G7**	**G8**
G1	**(0.83,0.74)** ^**1**^	3.42	3.10	3.77	4.73	4.23	4.13	***2.39***
G2	2.93	**(0.54,1.42)**	3.18	4.02	***5.31***	4.49	4.31	2.95
G3	3.39	3.29	**(1.05,0.92)**	3.98	5.13	4.25	4.35	2.67
G4	3.06	2.72	4.03	**(1.38,1.07)**	3.80	3.53	2.95	3.28
G5	3.94	3.94	***4.91*** ^***3***^	3.70	**(1.37,1.12)**	4.53	4.65	4.54
G6	2.67	2.80	3.63	2.61	4.09	**(1.14,NA** ^**2**^ **)**	4.15	3.41
G7	3.13	2.78	3.99	3.00	4.14	2.73	**(1.16,0.87)**	3.66
G8	2.29	2.53	3.08	2.75	4.16	***2.27***	2.73	**(0.87, 0.89)**

^*^Lower matrix; the partial 444 bp of gp51. Upper matrix; the complete gp51 gene.

^1^The values in bold along the diagonal are the average percentage within-genotype divergence (left, partial 444 bp of gp51; right, complete gp51 gene).

^2^NA denotes that calculation is not available due to a single data.

^3^Maximum and minimum divergences are represented by bold italic numbers for each dataset.

More divergent isolate sequences were observed in genotype 2 (from Argentina, Brazil, and Japan), with an average at 1.42% for the complete gp51 sequence, whereas genotype 4 included diverse isolates at 1.38% for the partial 444 bp of gp51 (Table 3, diagonal line). Genotype 4 was found in 9 out of 20 countries, including Argentina, Chile, Belarus, Russia, Ukraine, Poland, Belgium, France, and Germany, and the nucleotide variation within the genotype varied from 0% to 2.79% (Figure 1, Tables 2 and 3). The average genetic distance between genotypes did not exceed 5% in either datasets, except for G5 to G3 (5.13%) and G5 to G2 (5.31%) for the complete gp51 sequence (Table 3). In general, genetic variation within genotype 1 appeared to be minimal compared to that of other genotypes.

### Amino acid changes in the complete gp51 sequence

Eighty-seven deduced full-length amino acid sequences of BLV gp51 were aligned with the annotation of epitopes and functional domains as previously described [39,53-57] (Table 4). Specifically, amino acid sequences from the Korean BLV isolates were compared to 87 BLV isolates found throughout the world. Various single amino acid changes caused by a point mutation were discovered over the full length of gp51. A total of 85 amino acid variations were observed among the 301 amino acid sites, and 40 of these (47%) were observed in multiple isolates with at least two types of amino acids. Table 4 represents the distribution of the amino acid substitutions based on the parsimony-informative sites, according to genotype. Significant amino acid changes were not observed in the majority of Korean BLV isolates from genotype 1 compared to other isolates from different countries. However, it is interesting that only two isolates, GNCN-10 and 11, have 3 amino acid substitutions at 12 (Q- > P), 23 (T- > A), and 28 (C- > S), located in the leader peptide region, and 2 changes at 54 (K- > Q) and 69 (S- > L), corresponding to the conformational epitope region. In particular, one substitution at 140 (V- > A) in GNCN-1, GNCN-2, and GNCN-3 from Gyeongsangnam-do, occurs in the region of the second neutralizing domain (ND2), which is a binding zinc peptide that interacts with zinc ions and viral fusion proteins [58]. In addition, two isolates, GBGS-6 and 7, in Gyeongsangbuk-do had another amino acid change at 134 (D- > N) in the same region. Additionally, the Korean genotype 3 isolates were identical with those from Japan and the USA, which are based on amino acid substitutions. Interestingly, amino acid substitutions were more highly variable in the regions containing the conformational epitope region (Table 4). These changes were manly found in genotypes 4, 5, 6, and 7, encompassing European, Russian, and South American isolates. Additionally, some of the amino acid changes had a trend towards geographic clustering; for instance, substitutions found at 73 (A- > P) and 121 (R- > H) were only found in Russian and European isolates and substitutions at 15 (I- > V) and 37 (S- > T) were only found in Costa Rican isolates.

**Table 4:** **Amino acid alignment of the gp51 sequences from 87 isolates based on parsimony-informative sites**

		**LEADERR PEPTIDE** ^**2**^									**CONFORMATIONAL EPITOPE REGIONS** ^**3**^													**ND2** ^**4**^						**T** ^**5**^		**E-B** ^**6**^		**E-D** ^**7**^			**E-A** ^**8**^				
**Genotype**	**BLV isolates** ^**1**^																					1	**1**	**1**	**1**	**1**	**1**	**1**	**1**	**1**	**1**	**2**	**2**	**2**	**2**	**2**	**2**	**2**	**2**	**3**	**3**
			**1**	**1**	**2**	**2**	**2**	**2**	**2**	**3**	**3**	**4**	**5**	**5**	**5**	**6**	**6**	**7**	**7**	**8**	**8**	**2**	**3**	**3**	**3**	**4**	**4**	**4**	**4**	**8**	**8**	**2**	**3**	**5**	**6**	**7**	**9**	**9**	**9**	**0**	**0**
		**7**	**2**	**5**	**3**	**4**	**5**	**8**	**9**	**1**	**7**	**8**	**4**	**6**	**8**	**0**	**9**	**3**	**4**	**0**	**2**	**1**	**2**	**3**	**4**	**0**	**1**	**4**	**6**	**1**	**9**	**9**	5	**4**	**7**	**0**	**0**	**1**	**3**	**0**	**1**
G1	**Korea (Lim, 2009)** [24] **AY995174**	**S**	**Q**	**I**	**T**	**L**	**L**	**S**	**R**	**I**	**S**	**A**	**K**	**S**	**S**	**D**	**S**	**A**	**K**	**L**	**S**	**R**	**Q**	**A**	**D**	**A**	**N**	**I**	**F**	**Q**	**S**	**Y**	**S**	**S**	**R**	**F**	**T**	**A**	**S**	**R**	**R**
	***Korea (GBGS-1,2014) KP201466***							***C***																		***V***															
	***Korea (GBGS-2,2014) KP201463***							***C***																		***V***															
	***Korea (GBGS-3,2014) KP201460***																***L***									***V***															
	***Korea (GBGS-4,2014) KP201468***						***F***	***C***																		***V***															
	***Korea (GBGS-6,2014) KP201461***							***C***																	***N***	***V***															
	***Korea (GBGS-7,2014) KP201462***																								***N***	***V***															
	***Korea (GBGS-10,2014) KP201467***							***C***	*.*													***L***				***V***															
	***Korea (GNCN-1,2014) KP201474***							***C***	*.*																																
	***Korea (GNCN-2,2014) KP201472***							***C***	*.*																																
	***Korea (GNCN-3,2014) KP201480***							***C***																																	
	***Korea (GNCN-6,2014) KP201478***							***C***																		***V***															
	***Korea (GNCN-8,2014) KP201481***							***C***																		***V***													***F***		
	***Korea (GNCN-9,2014) KP201479***							***C***		***T***													***R***			***V***															
	***Korea (GNCN-10,2014) KP201475***		***P***		***A***								***Q***				***L***									***V***															
	***Korea (GNCN-11,2014) KP201482***		***P***		***A***								***Q***				***L***									***V***															
	Japan (Sagata,1985) [56] K02120							C																		V															
	Japan (Zhao, 2007) [30] EF065651							C																		V															
	Japan (Zhao, 2007) [30] EF065652						F	C	Q																	V															
	Japan (Zhao, 2007) [30] EF065653																									V															
	Japan (Zhao, 2007) [30] EF065657							C																		V															
	Japan (Zhao, 2007) [30] EF065658							C								N										V															
	Japan (Zhao, 2007) [30] EF065659							C								N										V				R											
	Japan (Zhao, 2007) [30] EF065660							C																		V															
	Japan (Zhao, 2007) [30] EF065661							C													F			T		V															
	Japan (Zhao, 2007) [30] EF065662							C																		V				R											
	Japan (Mekata, unpublished) LC007977^*^							C																		V					R										
	Japan (Mekata,unpublished) LC007978^*^							C																		V					R										
	Japan (Mekata,unpublished) LC007979^*^	P						C																		V					R										
	Japan (Mekata, unpublished) LC007980^*^							C																		V					R										
	Japan (Mekata,unpublished) LC007981^*^							C																		V					R										
	Japan (Mekata, unpublished) AB934282^*^							C																		V					R										
	Japan (Mekata, unpublished) LC007983^*^							C																		V															G
	Japan (Mekata, unpublished) LC007985^*^							C								N										V															
	Japan (Mekata,unpublished) LC007987^*^							C																		V															
	Japan (Mekata, unpublished) LC007988^*^							C																		V															
	Japan (Mekata, unpublished) LC007989^*^			T				C																T		V															
	Japan (Mekata, unpublished) LC007990^*^																L									V															
	Japan (Mekata, unpublished) LC007991^*^																									V															
	Japan (Mekata,unpublished) LC007992^*^			T																						V															
	USA (Mamoun, 1990) [16] M35239							C																		V															
	USA (Mamoun, 1990) [16] M35242							C																		V															
	USA (Johnston,2002) [15] AY078387							C																		V															
	USA (Zhao, 2007) [30] EF065641							C																		V															
	USA (Zhao, 2007) [30] EF065642							C																		V															
	USA (Zhao, 2007) [30] EF065644							C																		V															
	Costa Rica (Zhao, 2007) [30] EF065637							C																		V															
	Costa Rica (Zhao, 2007) [30] EF065640							C																		V															
	Uruguay (Moratorio, 2010) [51] FM209468							C																	N	V															P
	Uruguay (Moratorio, 2010) [51] FM209469							C																	N	V				S											P
	Uruguay (Moratorio, 2010) [51] FM209471							C																	N	V				S											P
	Uruguay (Moratorio, 2010) [51] FM209475							C																	N	V				S											P
	Uruguay (Moratorio, unpublished) HE967301^*^							C																	N	V															
	Uruguay (Moratorio, unpublished) HE967303^*^							C														H			N	V				S											
	Brazil (Stancek, 2002) [60] AY151262							C																	N	V															
	Brazil (Stancek, 2002) [60] AF547184							C																		V						C									
	Brazil (Camargos, 2002) [32] AF399703					V		C	Q												F				N	V															
	Australia (Coulston, 1990) [25] D00647		E					C	Q																	V															
**G3**	***Korea (GBGS-11,2014)KP201464***							***C***	***Q***												***F***			***T***		***V***	***D***							***L***							
	***Korea (GBGS-12,2014)KP201465***							***C***	***Q***												***F***			***T***		***V***	***D***							***L***							
	USA (Zhao, 2007) [30] EF65647							C	Q												F			T		V	D							L							
	USA (Zhao, 2007) [30] EF65648							C	Q												F			T		V	D							L							
	USA (Zhao, 2007) [30] EF65649							C	Q												F			T		V	D							L							
	Japan (Zhao, 2007) [30] EF65650							C	Q												F			T		V	D							L							
	Japan (Mekata, unpublished) LC007993^*^							C	Q												F			T		V	D							L							
**G2**	Argentina (Dube, 2000) [54] AF257515							C	Q												F					V	D							L		I	S	G	F	K	
	Argentina (Dube, 2009) [53] FJ914764							C	Q	T											F					V	D							L		I				K	
	Brazil (Camargos, 2002) [32] AF399704							C	Q												F					V	D							L							
**G8**	Russia (Lomakina, unpublished) JQ675760^*^							C	P												F					V								L	K						
	Russia (Lomakina, unpublished) JQ675759^*^							C	P												F					V								L	K						
**G6**	Brazil (Camargos, unpublished) AY185360*		E					C	Q			T							R		F					V		T						L	K**.**	I	L				
**G4**	Russia (Lomakina, unpublished) JQ686089*							C	Q			T						P	R		F	H				V							G	L							
	Russia (Lomakina,unpublished) JQ686093*							C	Q			T		F				P	R		F	H				V		T						L							
	Russia (Lomakina, unpublished) JQ686111*							C	Q			T		F				P	R		F	H				V		T						L							
	Belgium (Willems, 1993) [57] AF503581							C	Q			T			A			P	R		F	H				V							N	L							
	Belgium (Zhao, 2007) [30] EF065638	F						C	Q			T						P	R	S	F	H				V								L							
	Belgium (Mamoun, 1990) [16] M35240							C	Q			T			A			P	R		F	H				V							N	L							
	France (Mamoun, 1990) [16] M35238							C	Q			T		F				P	R		F	H				V		T						L							
	Poland (Mikiewicz, unpublished) AF067081^*^							C	Q			T						P	R		F	H				V								L							
**G7**	Russia (Lomakina, unpublished) JN695880^*^	P						C	Q			T							R		F					V								L				V			
	Russia (Lomakina, unpublished) JQ686120^*^	P						C	Q			T							R		F					V								L				V			
	Russia (Lomakina, unpublished) JQ675757^*^							C	Q			I							R		F					V			S					L				V			
	Moldova (Pluta, unpublished) KF801457^*^							C	Q			I							R		F					V	H							L				V			
	Moldova (Pluta,unpublished) KF801458^*^							C	Q			I							R		F					V						C		L				V			
**G5**	Costa Rica (Zhao, 2007) [30] EF065639			V		V		C			T	T							R	S	F		R	T		V								L			M	T			
	Costa Rica (Zhao, 2007) [30] EF065645			V		V		C	Q		T	T							R	S	F		R			V								L							
	Costa Rica (Zhao, 2007) [30] EF065654			V		V		C	Q		T	T							R	S	F		R			V								L			M	T			

*The Korean strain AY995174 was used as a template to verify amino acid substitutions from BLV sequences found throughout the world. The names of Korean isolates are marked in bold. The dots show homology areas with the template sequence, while amino acid changes are as indicated.

^1^All isolates were described in the following way: Korean BLV (names, isolation year) and reference BLV (the first author’s name, publication year or unpublished) [reference number] accession number. Unpublished data were marked with an asterisk.

^2-7^Leader peptide sites (7-31), conformational epitope regions (37-146), second neutralizing domain (ND2) (133-146), CD8^+^-T epitope (T) (181), various immune-stimulatory epitopes (E) B (229-235), D (254-270), A (290-293), and transmembrane hydrophobic regions (TMHR) (270-293) are shown by amino acid positions previously described in Moratorio *et al.* [39].

## Discussion

The phylogenetic analysis presented in this study supports previously established data indicating that BLV has eight genotype groups. We also discovered unknown phylogenetic relationships between newly analyzed sequences (i.e., more recently unpublished sequences from 2014), including isolates from Korea. Due to the different number of isolates for each genotype and two different gp51 regions sequenced for the two datasets (i.e., 903 bp and 444 bp), the phylogenetic results and genetic divergence values for the isolates are not the same in all analyses. Nevertheless, the eight genotypes of BLV were completely identified through comprehensive phylogenetic analyses. Our results are consistent with previous studies [17,18].

Maximum likelihood and Bayesian approaches showed that a complex of several BLV genotypes has worldwide distribution, and phylogenetic separation of the eight major genotypes was well resolved by all of the analyses. Additionally, we found that the phylogenetic relationships among the genotypes are correlated to the geographical origin of the isolates (e.g., G4-G7 in Europe/Russia, G5-G6 in South America) based on the complete gp51 sequence. As shown in Figures 1 and 2, genotype 1 is the most dominant BLV type in the world and is found in Australia, Iran, USA, Argentina, Brazil, Uruguay, Costa Rica, Japan, and Germany. Genotype 3 included only isolates from three geographically distinct countries, Japan, the USA, and Korea. Similarly, genotypes 5 and 6 were exclusively found in three countries, Costa Rica, Argentina, and Brazil in Central and South America. In addition, isolates of genotypes 8 and 7 mostly originated from Eurasian areas, excluding Korea and Japan. Noticeably, genotypes 1 and 4 covered large geographic areas from Europe to America, suggesting the possibility of extensive cattle trading between countries (Table 2).

Phylogenetic analyses of the complete gp51 gene revealed that 17Korean BLV isolates belonged to genotypes 1 and 3 (Additional file 1: Figure S1 and S2, Additional file 1: Figure S1). Seven genotype 1 isolates were found in Gyeongsangbuk-do (GBGS) and 8 isolates were found in Gyeongsangnam-do (GNCN). Indeed, genotype 1 was reported in Korea in 2009 [24] and was expected to be the dominant form found in our study. Two isolates from the Gyeongsangbuk-do region were assigned to genotype 3, a result that was also seen in a recent Japanese study [36]. Considering the geographic affinity of the two countries, these results are not unusual.

As shown in Table 3, the average distance between genotype 1 isolates (0.74% for the complete gp51 sequence and 0.83% for the partial gp51 sequence) was relatively lower than those observed within G2-G8. The low level of genetic diversity did not reflect geographic barriers, but perhaps indicates a single origin of BLV infection or international importation of cattle across the border from several countries.

Analyses of the BLV gp51 amino acid sequences in isolates collected from multiple geographic locations showed specific sequence conservation depending on the genotype (Table 4). Considering this result, a few nucleotide mutations or amino acid substitutions may affect the ability of BLV to survive and may infect hosts. The gp51 region characterized by amino acid changes of several functional sites remained unchanged over a long period in this study (e.g., 216/301 conserved region (72%)) (Table 4). Based on Korean genotype 1 BLV isolates, several amino acid changes were observed at positions 12, 23, and 28 in the leader peptide region and at positions 54 and 69 in the conformational epitope region in only two isolates from the GNCN locality of Gyeongsangnam-do. In addition, unique antigenic variations were identified in the second neutralizing domain, position 140 of GNCN-1, 2, and 3 along with position 134 of GBGS-6 and 7. The biological significance of these changes in the Korean isolates needs to be investigated by functional studies in *vivo*, but the changes in the conformational epitope overlapping with the second neutralizing domain region indicate a possibility of them affecting viral infectivity and formation.

## Conclusion

This study shows that two genotypes of BLV are circulating throughout Korea. It is notable that most of these isolates belong to genotype 1 (46 out of 50 isolates), which is referred to as the dominant BLV genotype around the world, whereas only 4 isolates belong to genotype 3. Further study to identify the diversity of BLV circulating in Korea is necessary with samples collected nationwide because we identified genotype 3 in Korean isolates. Two distinct genotypes imply that there were two independent introduction events in Korea. At this point, the exact geographic origin of the Korean isolates remains uncertain, even though they are highly similar (99%) to Costa Rican (EF065640) and Japanese (LC007985) genotype 1 isolates and highly similar (99%) to Japanese (EF065650) and USA (EF065648) genotype 3 isolates.

## Additional file

Additional file 1:
**Figure S1.** A Bayesian inference phylogenetic tree based on the partial gp51 (444 bp) sequences from different geographic regions. Korean isolates are shown in blue bold-italic names. The remaining isolates in the tree are denoted by country of origin, author with published date (or direct submitted date), and accession number. Genotypes 1 through 8 are indicated by vertical lines with the symbol ‘G’. Two numbers at the branches indicate BI posterior probabilities values (MrBayes(0–1)/Phycas(0–100)). An asterisk indicates unpublished or direct submission to GenBank sequences. + denotes that the selected sequences were used for the complete gp51 analyses. *Means that the sequences have not yet been investigated by a published phylogenetic analysis. The tree is rooted on genotype 5. **Table S1.** Accession number and geographic information regarding the fifty Korean BLV isolate sequences used in this study.

## References

[1] Burny A, Cleuter Y, Kettmann R, Mammerickx M, Marbaix G, Portetelle D (1988). Bovine leukaemia: facts and hypotheses derived from the study of an infectious cancer. Vet Microbiol.

[2] Kettmann R, Cleuter Y, Mammerickx M, Meunier-Rotival M, Bernardi G, Burny A (1980). Genomic integration of bovine leukemia provirus: comparison of persistent lymphocytosis with lymph node tumor form of enzootic. Proc Natl Acad Sci U S A.

[3] Kettmann R, Portetelle D, Mammerickx M, Cleuter Y, Dekegel D, Galoux M (1976). Bovine leukemia virus: an exogenous RNA oncogenic virus. Proc Natl Acad Sci U S A.

[4] Mirsky ML, Olmstead CA, Da Y, Lewin HA (1996). The prevalence of proviral bovine leukemia virus in peripheral blood mononuclear cells at two subclinical stages of infection. J Virol.

[5] Acaite J, Tamosiunas V, Lukauskas K, Milius J, Pieskus J (2007). The eradication experience of enzootic bovine leukosis from Lithuania. Prev Vet Med.

[6] Alfonso R, Almansa JE, Barrera JC (1998). [Serological prevalence and evaluation of the risk factors of bovine enzootic leukosis in the Bogota savannah and the Ubate and Chiquinquira Valleys, Colombia]. Rev Sci Tech.

[7] D’Angelino JL, Garcia M, Birgel EH (1998). Epidemiological study of enzootic bovine leukosis in Brazil. Trop Anim Health Prod.

[8] Jacobs RM, Pollari FL, McNab WB, Jefferson B (1995). A serological survey of bovine syncytial virus in Ontario: associations with bovine leukemia and immunodeficiency-like viruses, production records, and management practices. Can J Vet Res Revue Canadienne de Recherche Veterinaire.

[9] Marin C, de Lopez NM, Alvarez L, Lozano O, Espana W, Castanos H (1978). Epidemiology of bovine leukemia in Venezuela. Annales de Recherches Veterinaires Ann Vet Res.

[10] Murakami K, Kobayashi S, Konishi M, Kameyama K, Yamamoto T, Tsutsui T (2011). The recent prevalence of bovine leukemia virus (BLV) infection among Japanese cattle. Vet Microbiol.

[11] Nuotio L, Rusanen H, Sihvonen L, Neuvonen E (2003). Eradication of enzootic bovine leukosis from Finland. Prev Vet Med.

[12] Trono KG, Perez-Filgueira DM, Duffy S, Borca MV, Carrillo C (2001). Seroprevalence of bovine leukemia virus in dairy cattle in Argentina: comparison of sensitivity and specificity of different detection methods. Vet Microbiol.

[13] VanLeeuwen JA, Keefe GP, Tremblay R, Power C, Wichtel JJ (2001). Seroprevalence of infection with Mycobacterium avium subspecies paratuberculosis, bovine leukemia virus, and bovine viral diarrhea virus in maritime Canada dairy cattle. Can Vet J La Revue Veterinaire Canadienne.

[14] Wang CT (1991). Bovine leukemia virus infection in Taiwan: epidemiological study. J Vet Med Sci Jpn Soc Vet Sci.

[15] Johnston ER, Albritton LM, Radke K (2002). Envelope proteins containing single amino acid substitutions support a structural model of the receptor-binding domain of bovine leukemia virus surface protein. J Virol.

[16] Mamoun R, Morisson M, Rebeyrotte N, Busetta B, Couez D, Kettmann R (1990). Sequence variability of bovine leukemia virus env gene and its relevance to the structure and antigenicity of the glycoproteins. J Virol.

[17] Balic D, Lojkic I, Periskic M, Bedekovic T, Jungic A, Lemo N (2012). Identification of a new genotype of bovine leukemia virus. Arch Virol.

[18] Rola-Luszczak M, Pluta A, Olech M, Donnik I, Petropavlovskiy M, Gerilovych A (2013). The molecular characterization of bovine leukaemia virus isolates from Eastern Europe and Siberia and its impact on phylogeny. PLoS One.

[19] Choi W (1982). Survey for antibodies to bovine leukemia virus in dairy and Korean native cattle. Korean J Vet Res.

[20] Suh G, Lee C, Lee C, Hur T, Kang S, Son D (2003). Prevalence of anti-bovine leukemia virus antibodies in dairy and Korean native cattle. J Vet Clin.

[21] Jung K, Shim H, Baek J (2012). Investigation of bovine leukemia virus infection in dairy farms of northern Gyeonggi province, Korea. Korean J Vet Serv.

[22] Suh G, Lee J, Lee C, Hur T, Son D, Ahn B (2005). Establishment of a bovine leukemia virus-free dairy herd in Korea. J Vet Sci.

[23] Chu K, Hyong S, Im J, Seo L (2007). Seroprevalence of infection with Neospora caninum, Mycobacterium paratuberculosis, bovine leukosis and Brucella abortus of dairy cattle in Jeonbuk-Iksan area. Korean J Vet Serv.

[24] Lim S, Jeong W, Tark D, Yang D, Kweon C (2009). Agar gel immunodiffusion analysis using baculovirus-expressed recombinant bovine leukemia virus envelope glycoprotein (gp51/gp30T-). J Vet Sci.

[25] Coulston J, Naif H, Brandon R, Kumar S, Khan S, Daniel RC (1990). Molecular cloning and sequencing of an Australian isolate of proviral bovine leukaemia virus DNA: comparison with other isolates. General Virol.

[26] Felmer R, Munoz G, Zuniga J, Recabal M (2005). Molecular analysis of a 444 bp fragment of the bovine leukaemia virus gp51 env gene reveals a high frequency of non-silent point mutations and suggests the presence of two subgroups of BLV in Chile. Vet Microbiol.

[27] Hemmatzadeh F (2007). Sequencing and phylogenetic analysis of gp51 gene of bovine leukaemia virus in Iranian isolates. Vet Res Commun.

[28] Molteni E, Agresti A, Meneveri R, Marozzi A, Malcovati M, Bonizzi L (1996). Molecular characterization of a variant of proviral bovine leukaemia virus (BLV). Zentralblatt fur Veterinarmedizin Reihe B J Vet Med Series B.

[29] Monti G, Schrijver R, Beier D (2005). Genetic diversity and spread of Bovine leukaemia virus isolates in Argentine dairy cattle. Arch Virol.

[30] Zhao X, Buehring GC (2007). Natural genetic variations in bovine leukemia virus envelope gene: possible effects of selection and escape. Virology.

[31] Camargos MF, Pereda A, Stancek D, Rocha MA, dos Reis JK, Greiser-Wilke I (2007). Molecular characterization of the env gene from Brazilian field isolates of Bovine leukemia virus. Virus Genes.

[32] Camargos MF, Stancek D, Rocha MA, Lessa LM, Reis JK, Leite RC (2002). Partial sequencing of env gene of bovine leukaemia virus from Brazilian samples and phylogenetic analysis. Vet Med B Infect Dis Vet Publ Health.

[33] Rodriguez SM, Golemba MD, Campos RH, Trono K, Jones LR (2009). Bovine leukemia virus can be classified into seven genotypes: evidence for the existence of two novel clades. J General Virol.

[34] Licursi M, Inoshima Y, Wu D, Yokoyama T, González ET, Sentsui H (2003). Provirus variants of bovine leukemia virus in naturally infected cattle from Argentina and Japan. Vet Microbiol.

[35] Asfaw Y, Tsuduku S, Konishi M, Murakami K, Tsuboi T, Wu D (2005). Distribution and superinfection of bovine leukemia virus genotypes in Japan. Arch Virol.

[36] Matsumura K, Inoue E, Osawa Y, Okazaki K (2011). Molecular epidemiology of bovine leukemia virus associated with enzootic bovine leukosis in Japan. Virus Res.

[37] Fechner H, Blankenstein P, Looman AC, Elwert J, Geue L, Albrecht C (1997). Provirus variants of the bovine leukemia virus and their relation to the serological status of naturally infected cattle. Virology.

[38] Katoh K, Standley DM (2013). MAFFT multiple sequence alignment software version 7: improvements in performance and usability. Mol Biol Evol.

[39] Moratorio G, Fischer S, Bianchi S, Tomé L, Rama G, Obal G (2013). A detailed molecular analysis of complete bovine leukemia virus genomes isolated from B-cell lymphosarcomas. Vet Res.

[40] Kimura M (1980). A simple method for estimating evolutionary rate of base substitutions through comparative studies of nucleotide sequences. J Mol Evol.

[41] Tamura K, Peterson D, Peterson N, Stecher G, Nei M, Kumar S (2011). MEGA5: molecular evolutionary genetics analysis using maximum likelihood, evolutionary distance, and maximum parsimony methods. Mol Biol Evol.

[42] Darriba D, Taboada GL, Doallo R, Posada D (2012). jModelTest 2: more models, new heuristics and parallel computing. Nat Methods.

[43] Zwickl DJ (2006). Genetic algorithm approaches for the phylogenetic analysis of large biological sequence datasets under the maximum likelihood criterion [Dissertation].

[44] Stamatakis A (2006). RAxML-VI-HPC: maximum likelihood-based phylogenetic analyses with thousands of taxa and mixed models. Bioinformatics.

[45] Silvestro D, Michalak I (2011). raxmlGUI: a graphical front-end for RAxML. Org Divers Evol.

[46] Sukumaran J, Holder MT (2010). DendroPy: a Python library for phylogenetic computing. Bioinformatics.

[47] Ronquist F, Teslenko M, van der Mark P, Ayres DL, Darling A, Hohna S (2012). MrBayes 3.2: efficient Bayesian phylogenetic inference and model choice across a large model space. Syst Biol.

[48] Yang Z, Rannala B (2005). Branch-length prior influences Bayesian posterior probability of phylogeny. Syst Biol.

[49] Phycas 1.2.0 [http://www.phycas.org]

[50] Lewis PO, Holder MT, Holsinger KE (2005). Polytomies and Bayesian phylogenetic inference. Syst Biol.

[51] Moratorio G, Obal G, Dubra A, Correa A, Bianchi S, Buschiazzo A (2010). Phylogenetic analysis of bovine leukemia viruses isolated in South America reveals diversification in seven distinct genotypes. Arch Virol.

[52] D’Angelino RH, Pituco EM, Villalobos EM, Harakava R, Gregori F, Del Fava C (2013). Detection of bovine leukemia virus in brains of cattle with a neurological syndrome: pathological and molecular studies. BioMed Res Int.

[53] Dube S, Abbott L, Dube DK, Dolcini G, Gutierrez S, Ceriani C (2009). The complete genomic sequence of an in vivo low replicating BLV strain. Virol J.

[54] Dube S, Dolcini G, Abbott L, Mehta S, Dube D, Gutierrez S (2000). The complete genomic sequence of a BLV strain from a Holstein cow from Argentina. Virology.

[55] Rice NR, Stephens RM, Couez D, Deschamps J, Kettmann R, Burny A (1984). The nucleotide sequence of the env gene and post-env region of bovine leukemia virus. Virology.

[56] Sagata N, Yasunaga T, Tsuzuku-Kawamura J, Ohishi K, Ogawa Y, Ikawa Y (1985). Complete nucleotide sequence of the genome of bovine leukemia virus: its evolutionary relationship to other retroviruses. Proc Natl Acad Sci U S A.

[57] Willems L, Thienpont E, Kerkhofs P, Burny A, Mammerickx M, Kettmann R (1993). Bovine leukemia virus, an animal model for the study of intrastrain variability. J Virol.

[58] Gatot JS, Callebaut I, Van Lint C, Demonte D, Kerkhofs P, Portetelle D (2002). Bovine leukemia virus SU protein interacts with zinc, and mutations within two interacting regions differently affect viral fusion and infectivity in vivo. J Virol.

[59] Derse D, Diniak AJ, Casey JW, Deininger PL (1985). Nucleotide sequence and structure of integrated bovine leukemia virus long terminal repeats. Virology.

[60] Stancek D, Camargos MF, Reis JKP, Rocha MA, Lessa LM, Leite FSC, et al. Higher frequency of non-silent point mutations in some bovine leukemia virus (BLV) strains isolated in Brazil in comparison with the strains circulating in several other countries. J Vet Med B Infect Dis Vet Public Health. 2002. In press 2002.

