# Supplementary material for: Sequencing and phylogenetic analysis of the gp51 gene from Korean bovine leukemia virus isolates
Source: Virol J. 2015 Apr 15;12:64. doi: 10.1186/s12985-015-0286-4 (PMC4405874; doi:10.1186/s12985-015-0286-4)
Supplement: Additional file 1: — Figure S1. A Bayesian inference phylogenetic tree based on the partial gp51 (444 bp) sequences from different geographic regions. Korean isolates are shown in blue bold-italic names. The remaining isolates in the tree are denoted by country of origin, author with published date (or direct submitted date), and accession number. Genotypes 1 through 8 are indicated by vertical lines with the symbol ‘G’. Two numbers at the branches indicate BI posterior probabilities values (MrBayes(0–1)/Phycas(0–100)). An asterisk indicates unpublished or direct submission to GenBank sequences. + denotes that the selected sequences were used for the complete gp51 analyses. *Means that the sequences have not yet been investigated by a published phylogenetic analysis. The tree is rooted on genotype 5. Table S1. Accession number and geographic information regarding the fifty Korean BLV isolate sequences used in this study. [file 12985_2015_286_MOESM1_ESM.doc]

**Figure S1. A** Bayesian inference phylogenetic tree based on the partial gp51 (444 bp) sequences from different geographic regions. Korean isolates are shown in blue bold-italic names. The remaining isolates in the tree are denoted by country of origin, author with published date (or direct submitted date), and accession number. Genotypes 1 through 8 are indicated by vertical lines with the symbol ‘G’. Two numbers at the branches indicate BI posterior probabilities values (MrBayes(0–1)/Phycas(0–100)). An asterisk indicates unpublished and direct submission to GenBank sequences. + denotes that the selected sequences were used for the complete gp51 analyses. * means that the sequences have not yet been investigated by a published phylogenetic analysis. The tree is rooted on genotype 5.

**Table S1.** Accession number and geographic information regarding the fifty Korean BLV isolate sequences used in this study.

| No. | Accession | Geographic origin | ID | Genotype |
| --- | --- | --- | --- | --- |
| 1 | KP201460 | Gyeongsangbuk-do | GBGS-3 | 1 |
| 2 | KP201461 |  | GBGS-6 | 1 |
| 3 | KP201462 |  | GBGS-7 | 1 |
| 4 | KP201463 |  | GBGS-2 | 1 |
| 5 | KP201464 |  | GBGS-11 | 3 |
| 6 | - |  | - | 3 |
| 7 | - |  | - | 3 |
| 8 | KP201465 |  | GBGS-12 | 3 |
| 9 | KP201466 |  | GBGS-1 | 1 |
| 10 | - |  | - | 1 |
| 11 | - |  | - | 1 |
| 12 | - |  | - | 1 |
| 13 | - |  | - | 1 |
| 14 | - |  | - | 1 |
| 15 | KP201467 |  | GBGS-10 | 1 |
| 16 | KP201468 |  | GBGS-4 | 1 |
| 17 | KP201469 |  | GBGS-8 | 1 |
| 18 | KP201470 |  | GBGS-9 | 1 |
| 19 | KP201471 |  | GBGS-5 | 1 |
|  |  |  |  |  |
| 20 | KP201472 | Gyeongsangnam-do | GNCN-2 | 1 |
| 21 | - |  | - | 1 |
| 22 | - |  | - | 1 |
| 23 | - |  | - | 1 |
| 24 | - |  | - | 1 |
| 25 | KP201473 |  | GNCN-7 | 1 |
| 26 | KP201474 |  | GNCN-1 | 1 |
| 27 | - |  | - | 1 |
| 28 | - |  | - | 1 |
| 29 | - |  | - | 1 |
| 30 | - |  | - | 1 |
| 31 | - |  | - | 1 |
| 32 | - |  | - | 1 |
| 33 | - |  | - | 1 |
| 34 | - |  | - | 1 |
| 35 | - |  | - | 1 |
| 36 | - |  | - | 1 |
| 37 | - |  | - | 1 |
| 38 | - |  | - | 1 |
| 39 | - |  | - | 1 |
| 40 | - |  | - | 1 |
| 41 | - |  | - | 1 |
| 42 | KP201475 |  | GNCN-10 | 1 |
| 43 | KP201476 |  | GNCN-4 | 1 |
| 44 | KP201477 |  | GNCN-5 | 1 |
| 45 | KP201478 |  | GNCN-6 | 1 |
| 46 | - |  | - | 1 |
| 47 | KP201479 |  | GNCN-9 | 1 |
| 48 | KP201480 |  | GNCN-3 | 1 |
| 49 | KP201481 |  | GNCN-8 | 1 |
| 50 | KP201482 |  | GNCN-11 | 1 |
